# Record Dynamics in Ants

**DOI:** 10.1371/journal.pone.0009621

**Published:** 2010-03-11

**Authors:** Thomas O. Richardson, Elva J. H. Robinson, Kim Christensen, Henrik J. Jensen, Nigel R. Franks, Ana B. Sendova-Franks

**Affiliations:** 1 Department of Mathematics and Statistics, Bristol Institute of Technology, University of the West of England, Bristol, United Kingdom; 2 School of Biological Sciences, University of Bristol, Bristol, United Kingdom; 3 Department of Biology and York Centre for Complex Systems Analysis, University of York, York, United Kingdom; 4 Institute for Mathematical Sciences, Imperial College London, London, United Kingdom; 5 Blackett Laboratory, Department of Physics, Imperial College London, London, United Kingdom; 6 Department of Mathematics, Imperial College London, London, United Kingdom; Vrije Universiteit, Netherlands

## Abstract

The success of social animals (including ourselves) can be attributed to efficiencies that arise from a division of labour. Many animal societies have a communal nest which certain individuals must leave to perform external tasks, for example foraging or patrolling. Staying at home to care for young or leaving to find food is one of the most fundamental divisions of labour. It is also often a choice between safety and danger. Here we explore the regulation of departures from ant nests. We consider the extreme situation in which no one returns and show experimentally that exiting decisions seem to be governed by fluctuating record signals and ant-ant interactions. A record signal is a new ‘high water mark’ in the history of a system. An ant exiting the nest only when the record signal reaches a level it has never perceived before could be a very effective mechanism to postpone, until the last possible moment, a potentially fatal decision. We also show that record dynamics may be involved in first exits by individually tagged ants even when their nest mates are allowed to re-enter the nest. So record dynamics may play a role in allocating individuals to tasks, both in emergencies and in everyday life. The dynamics of several complex but purely physical systems are also based on record signals but this is the first time they have been experimentally shown in a biological system.

## Introduction

Ant societies are shaped by selection that operates, in part, at the level of the colony [1], so the success of the individual is intimately bound to that of its colony. Outside-nest work is dangerous and the rate of attrition of outside-nest workers through predation or adverse environmental conditions is often high.

The life-cycles of ant societies are dominated by growth or decline [2]. Thus they are rarely at a steady state and are typically non-stationary. Here we induce non-stationarity by permanently eliminating all ants that exit the nest and compare these colonies with controls in which ants can freely leave and re-enter the nest. We use analytical methods developed for the analysis of out-of-equilibrium physical systems to explore the nature of the mechanism governing the decisions of individual ants to leave the nest. Indeed, biological systems are, like other systems in Nature, generically non-equilibrium systems since they are not isolated from external influences and continuously have a flux of mass or energy passing through them [3].

The null model for a system in which successive events are drawn from a diminishing pool is one with an exponentially declining event rate, as in radioactive decay. In this scenario there are either no interactions between the components or interactions between the components are not correlated with decay events. The simplest form of radioactive decay is one in which all the components have an identical decay probability, which can be modelled as a homogeneous Poisson process. Obviously ants are not all identical, so for our null model we implement a heterogeneous Poisson process by assuming that the component parts (the ants) vary in their decay (i.e. exit) probabilities.

An alternative scenario that produces rapidly decreasing event rates is one in which events are triggered when a fluctuating variable - the record signal - exceeds its historical ‘high water mark’. If the record signal fluctuates randomly, the increment between successive record values becomes progressively smaller and the rate at which new records accrue drops off according to the inverse of time [4], [5]. Hence the rate of change is a function of the age of the system. An intuitive example of a rapidly decelerating record time-series (albeit one that is probably not based on record signals as defined in complex systems) is the accumulation of human sporting records, where the rate at which new records accumulate depends largely on the age of the sport [6], [7]. All cases in which fluctuating record signals trigger events, include strong interactions between the component parts and involve long-range correlations that span the entire system [8], [9], [10], [11].

While exponential decay is characterised by Poisson statistics in linear time, record dynamics is characterised by Poisson statistics in logarithmic time [4], [5], [8], [12], [13], [14]. What mechanism can generate such log-Poisson statistics? A fluctuating record signal will only produce log-Poisson statistics if each successive value of the underlying fluctuating signal is independent of its predecessors. Independence of the fluctuating record signal leads the record times to be uncorrelated in logarithmic time, so the record value at time log (T_k_) is independent of previous records at time log (T_k-n_). Crucially, the distribution of the underlying fluctuating signal from which the record signal is derived, must not change over time. Quite remarkably, irrespective of the probability distribution of the underlying fluctuating signal, records will accrue at a logarithmically decreasing rate [5].

We test whether nest leaving activity is compatible with either of two models of rapidly decelerating events: exponential decay as a null model or record dynamics. We further test the effects of heterogeneous units and varying colony size on the exponential decay model through a simulation parameterised from data.

## Materials and Methods

### Experiments

Fifteen *T. albipennis* colonies were collected from rock crevices in Dorset, UK. They were housed in nests constructed from a cardboard cavity sandwiched between a pair of microscope slides, and maintained according to established protocols [15]. All colonies were queenright and had a complement of brood at various stages of development. The mean number of workers in each colony was 121 (median = 139, min = 73, max = 150, interquartile range = 59), the mean number of brood was 112 (median = 100, min = 56, max = 259, interquartile range = 68), and the mean worker:brood ratio was 1.30 (median = 1.16, min = 0.56, max = 2.48, interquartile range = 0.53). There is no evidence of a day-night cycle or regular periodicity of activity bouts in *T. albipennis* (Robinson *et al.* 2009). Colonies were maintained at a constant temperature (24°C), with continuous lighting.

In the treatment when ants were removed as they left the nest, the number remaining in the nest decreased. So to test the effect of external worker removals, the ‘events’ (ant exits) in the non-removal condition had also to be drawn from a diminishing pool of ants that could potentially leave the nest. Hence in the control an event was defined as the exit of a ‘new ant’, one *that had not been seen leaving the nest since the start of observation*.

The fifteen colonies were allocated to treatment and control groups as follows.

#### 60 hour non-removal control (colonies 1–7)

We recorded the times that previously-unseen ‘new ants’ first left the nest. Each nest was placed in an identical arena, in which food and water were available ad-lib. All the ants in seven colonies were fitted with passive RFID tags so that the time and identities of ants leaving the nest could be recorded by an RFID reader over the nest exit [16].

#### 12 hour removal treatment (colonies 1,2,5,6 & 7)

Five of the seven colonies fitted with passive RFID tags from the non-removal control were later used for external worker removal.

#### 2 hour removal treatment (colonies i–vi)

A different set of six colonies were subjected to a shorter 120 minute removal period. Every time an ant left the nest, it was removed and the time noted.

#### 5+ day removal treatment (colonies 8 & 9)

Two colonies were subjected to continuous worker removal lasting for 140 and 200 hours. Every time an ant walked to the nest exit, its movement was detected automatically by a high-resolution webcam (Logitech QC deluxe) positioned immediately above the exit and attached to a PC. When an ant was detected leaving the nest, the computer opened a valve, releasing a short burst of pressurised air resulting in the ant being expelled from the nest exit into a collection dish.

### A Null Model of Nest Leaving by ‘Weighting Waiting’

To test the exponential decay scenario further we consider a simple model that generates a rapidly decelerating exit rate using individual variation in intrinsic exit probabilities in which independent ‘decay’ events are equivalent to exits. In this scenario the declining exit rate is due to the diminishing number of ants, and because the fewer ants that remain, the lower the average exit probability per ant. No correlation between the ants will emerge since their decision to exit is entirely determined by processes referring to the individual ant. *Weighting Waiting* treats the observed exit time series as originating from a heterogeneous Poisson process which is similar to classical radioactive decay, but differs in two respects: the distribution of individual decay (exit) probabilities is heterogeneous and the number of components is finite. Ants carry food reserves in their gaster (the bulbous part of their abdomen). In *T. albipennis* corpulence is positively related to gaster dry-weight [17], which is itself positively related to waiting times between exits [16]. Thus we parameterised the exit probability distribution from the experimental distribution of gaster dry-weights (see Fig. S3 for gaster weight data). Individuals with a light gaster tend to exit the nest sooner than those with a heavier gaster, but an ant of *any* weight can potentially exit at any time, so the greater the weight the longer the wait.

At the start of each run of the simulation, every ‘ant’ in the colony was randomly assigned a gaster weight from the empirical distribution (Fig. S3), thus giving it an exit probability and creating individual heterogenity. In every time-step each ant decided whether to leave the nest by comparing its exit probability to a uniformly-distributed random number between 0 and 1. If the ant's exit probability was greater than this random number, the ant was deemed to have left the nest, and was eliminated from the simulation. We recorded the same statistics for these simulated ants, as for the real ants.

### Statistics

In a Poisson process, if T_k_ is the time of the k^th^ event, then the waiting times between events, *w* = T_k_-T_k-1_ have the exponential distribution P(*w*>x) = e^–λx^. Analogously, in a log-Poisson process the logarithmic waiting time, τ is equivalent to the difference between the logarithms of successive event times, which in turn is equal to the logged ratio of successive event times, *τ* = Ln(T_k_)-Ln(T_k-1_) = Ln(T_k_/T_k-1_), and has the exponential distribution P(*τ*>x) = e^–λx^. Therefore we test whether either the waiting time, *w* or the logarithmic waiting time, *τ* is exponentially distributed.

A major feature of the Poisson process is the absence of any correlation between events, so the duration of successive waiting times, *w*, should not be related. Similarly, a log-Poisson process will not display correlations in the sequence of logarithmic waiting times, τ. Therefore we test for the presence of correlations in the sequence of logarithmic waiting times.

Furthermore, in a Poisson process the average number of events, <N>, grows linearly over time, and similarly in a log-Poisson process <N> grows linearly over the logarithm of time, so <N*_t_*> = *α* Ln *t*, when *t*»1. Hence we test whether the number of ants leaving the nest is constant in logarithmic time, and compare this to the null model.

Finally, in systems that decelerate according to record dynamics, the average rate of events, *r*, decreases with the inverse of the system age, so, *r* ∝ *t* ^−1^ [4]. Thus we test if the rate of ants leaving the nest drops off in this manner, and compare the observed exit rate for the ants to that predicted by the null model.

## Results

**Figure 1:**
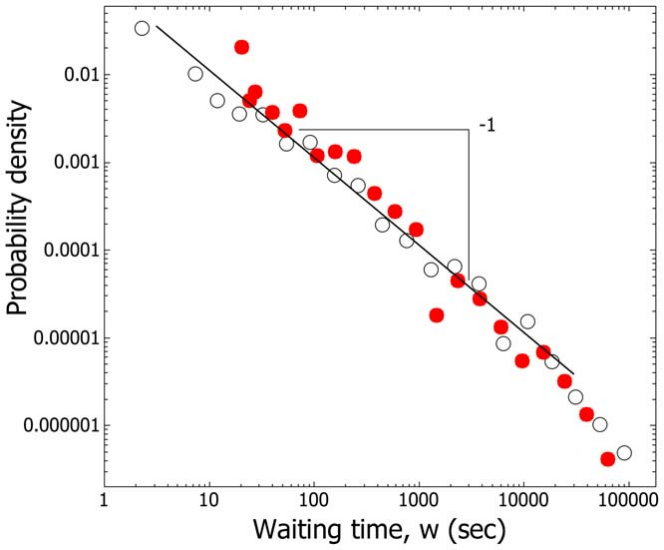
Waiting times between exits are not exponentially distributed. In both removal (○) and non-removal (•) conditions for the five colonies which underwent both the waiting time probability densities between exits, *w* = T_k_-T_k-1_, follow a heavy-tailed distribution, that is closer to a power-law distribution, P(*w*) = *w^−^* ^k^, where k = 1, than an exponential, P(*w*) = e^−λ*w*^, which will not give a straight line on a log-log plot.

**Figure 2:**
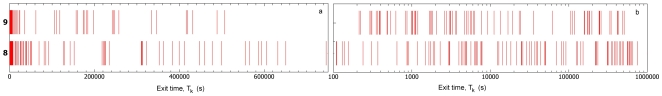
Events occur at random in logarithmic time. Graphical representation of ant exits in the removal condition. The rate of ant exits rapidly decelerates in time, and in logarithmic time the Poisson nature of events is evident. The red bars represent exits a) Untransformed data for the two longest time-series of ant removals; 5+ days, colonies 8 and 9, as indicated on the y-axis. b) The same data in logarithmic time, where the pattern matches what would be expected from a random (Poisson) process. After logarithmic transformation the ratios between sequential exit times, (T_k_) are exponentially distributed (see Fig. 3).

Ant exits were not compatible with the null model of exponential decay because the probability density of waiting times, *w*, is a straight line on a log-log plot and this is not consistent with an exponential distribution (Fig. 1). Rather, the temporal statistics are compatible with record dynamics. In logarithmic time the random (i.e. Poisson) structure of the exits is visible (Fig. 2).

**Figure 3:**
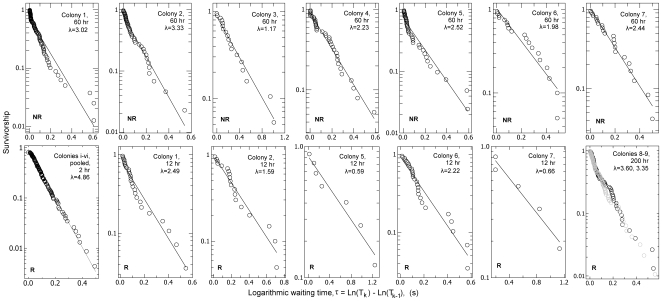
The logarithmic waiting time, τ, is exponentially distributed. Survivorship of *τ*, P(*τ*>*x*) where *τ* = ln (T_k_/T_k-1_), the logarithmic waiting time. The ordinate is logged as a straight line on such a plot indicates that event rate is independent of logged waiting time, i.e. that P(*τ*>x) = e^−λx^. The straight lines are least squares regressions (see Table S1). Column 1, row 2: Pooled *τ* distributions for colonies i-vi (see Fig. S1 for non-pooled plots).

**Figure 4:**
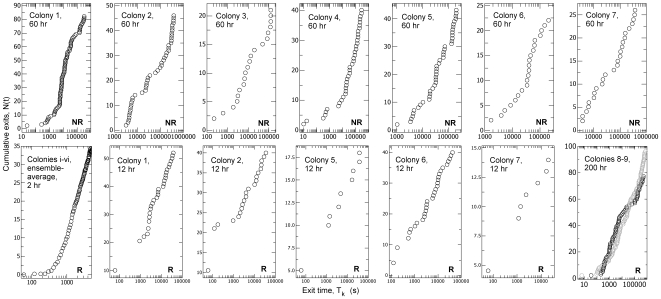
The cumulative number of exits increases linearly in logarithmic time. Accumulated number of ant exits over time, N(*t*) to exit time, T_k_. The abscissa is logged to check for constant exit rates in logarithmic time when T_k_≫1. NR = non-removal control, R = removal treatment. Colum 1, row 2; Ensemble average, <N(*t*)> for six colonies (i–vi) undergoing 2 hours of external worker removal.

**Figure 5:**
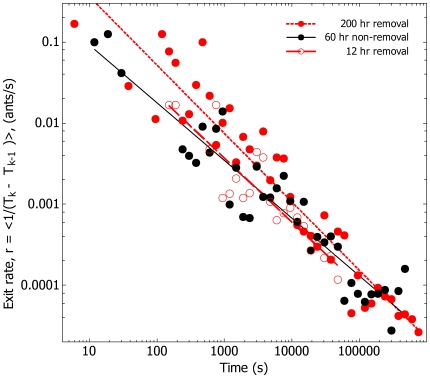
The event rate decreases as a function of the inverse of time, *r* ∝ *t* ^−1^. For both removal and non-removal, mean ant exit rates, (T_k_-T_k-1_)^−1^, are non-stationary and drop off according to the inverse of time. •; 60 hr non-removal, Log 10 (T_k_-T_k-1_)^−1^ = 0.19 - 0.71*Log 10 T_k_. ○; 12 hr removal, Log 10 (T_k_-T_k-1_)^−1^ = 0.31 - 0.79*Log 10 T_k_. •; 200 hr removal, Log 10 (T_k_-T_k-1_)^−1^ = 0.40–0.84*Log 10 T_k_.

**Figure 6:**
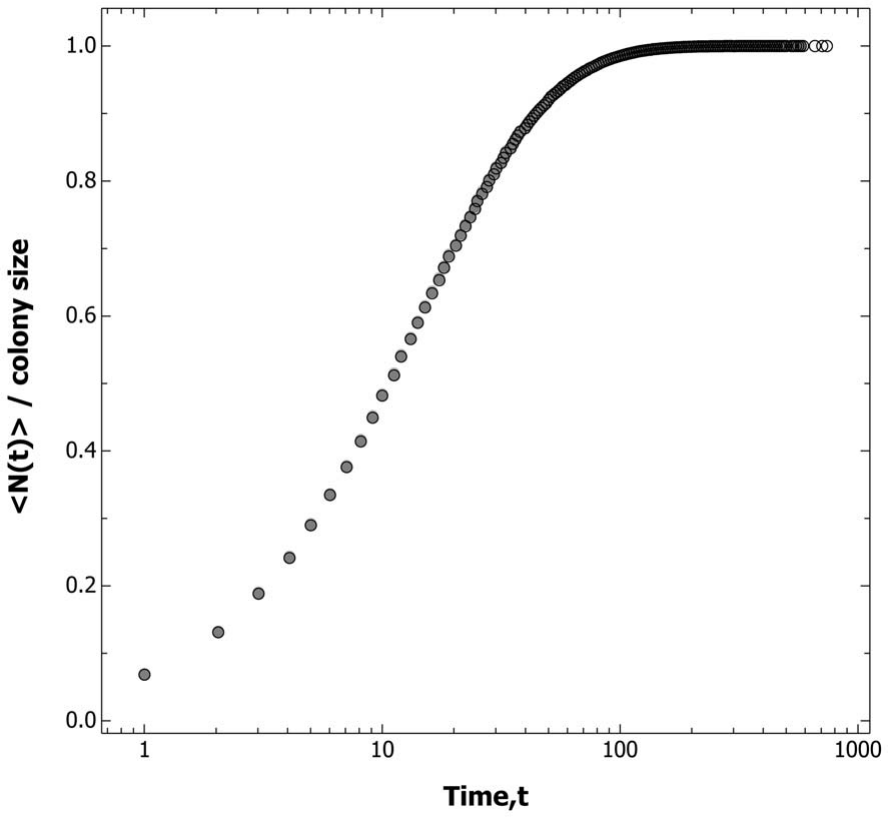
In the null model the accumulated number of events does not increase linearly in log time. Mean accumulated number of exits over time, <N(T_k_)> over logarithmic T_k_, with N(t) normalised by colony size (○; colony size = 500, •; 100, •; 50). Each colony size was run for 2×10^4^ independent realisations. The points for each colony size overlay each other. The finite-size effect inherent in our heterogeneous Poisson process model means that even when the ants drew their exit probabilities from a more right-skewed distribution than the empirical gaster weight distribution, the pattern of exits was qualitatively similar.

**Figure 7:**
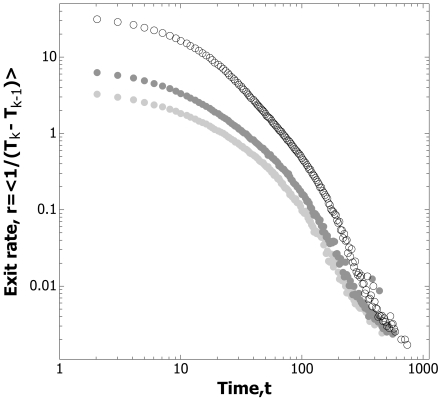
In the null model the event rate does not decrease as a function of the inverse of time. The mean event rate rate over time for the Weighting Waiting model. Same data as Fig. 6. (○; colony size = 500, •; 100, •; 50).

The evidence for record dynamics is as follows. For both control and treatment the logarithmic waiting time, that is, the logged ratio of successive exit times, *τ* = Ln(T_k_/T_k-1_), is compatible with an exponential distribution (Fig. 3, Text S1, Fig. S1). Additional evidence for compatibility with log-Poisson statistics is provided by the demonstration that the sequence of *τ* values is ‘memoryless’, that is there is little autocorrelation between successive logarithmic waiting times (Text S1, Fig. S2), as expected in a log-Poisson process [4]. Furthermore, the cumulative number of exits increases linearly over the logarithm of time, that is the event rate is constant in logarithmic time (Fig. 4). In both removal and non-removal conditions the rate of exits, *r*, decreases as an inverse function of time elapsed (Fig. 5), so *r* ∝ *t* ^−1^. By contrast, the cumulative number of events produced by the Weighting Waiting model increases as a sigmoid, rather than a linear function of the logarithm of time (Fig. 6), and the event rate does not fall off according to the inverse of the time elapsed (Fig. 7).

**Figure 8:**
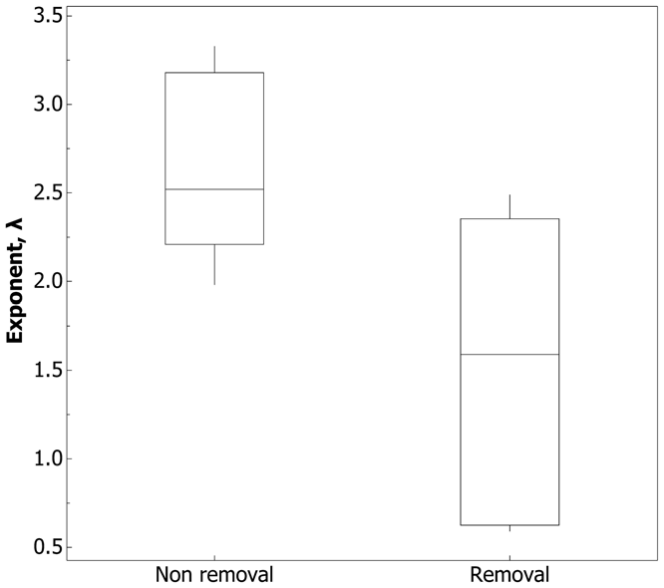
The exit rate is down-regulated when ants fail to return. The rate exponent, λ, from the *τ* distribution for five colonies that underwent both non-removal and removal conditions (colonies 1,2,5,6 & 7, Table S1). Smaller values of λ indicate the *τ* distribution is skewed towards bigger values of *τ*; t-test, t_8_ = 2.52, p = 0.036.

Although exits under both removal and non-removal conditions display qualitatively identical log-Poisson statistics, there are quantitative differences between them. The intensity exponent, λ, from P(*τ* >x) = e^–λx^ was significantly lower in the removal condition (Fig. 8, Table S1). Put another way, when ants are not allowed to return, the distribution of the logged ratio of successive waiting times, *τ* was more skewed towards larger values. This means ants took longer to exit the nest.

## Discussion

We found that ant exits were compatible with a record dynamics process while the null model could not reproduce the observed statistics. Therefore, the temporal pattern of ant exits implies that ant-ant interactions and fluctuating record signals govern individual nest-leaving decisions. Log-Poisson statistics are only found when the system is in a statistically non-stationary or ‘transient’ state [4], [5]. Thus the demonstration of record dynamics implies that the colony is not in a steady state, i.e. not at equilibrium, even when the population size is stationary. Such a mechanism might be adaptive for a dangerous task such as leaving the nest, because individuals may only do so when the demand reaches an unprecedented level, for example, the brood may be hungrier and the adult workforce thirstier than ever before.

Intriguingly, record dynamics were present both when ants were removed and in the non-removal control. In the treatment the colony population size was forced to decrease. Similarly, in the control the definition of an event as the exit of any ant previously not seen leaving the nest effectively creates a diminishing sub-population of individuals that have yet to be observed leaving the nest. This sub-population is the equivalent of the population remaining in the nest in the experiment where ants are prevented from returning, as it also undergoes a decline towards zero, when all ants in the colony would have been observed leaving the nest at least once. As the same qualitative pattern was found under both the removal and non-removal condition, interactions with ants that *have not* recently returned to the nest seem sufficient for the generation of log-Poisson statistics. So fluctuating record signals and ant-ant interactions may regulate individual exit decisions irrespective of whether external workers return to the nest.

If the temporal pattern of exits appears to be qualitatively identical under both benign and hostile environmental conditions, are there any quantitative differences? When workers are prevented from returning, the distribution of *τ* is significantly more skewed towards larger values (Fig. 8). In honeybees [18], bumblebees [19], and other ants [2], stimuli derived from interactions with returning individuals up-regulate the exit rate. However, to our knowledge this is the first evidence that the complete severance of this feedback loop results in down-regulation of the exit rate.

It should be emphasised that the statistical mechanism described in the high water-mark record-dynamics model, is a macroscopic *summary* of the processes occurring at the microscopic level. It *does not* describe the specific microscopic processes. However scenarios in which fluctuating record signals trigger events are always associated with long-range correlations that span the system. These correlations arise out of short-range interactions between the components [5], [10]. Since the designation of a new record depends on the preceding sequence of record values, and because through local interactions, the components share this collective history, all components involved in a process controlled by record dynamics must necessarily be strongly correlated [8], [9], [10], [11].

Intermittent dynamics associated with event rates that decelerate rapidly, but non-exponentially, have been described in 10000 generation experiments of bacterial evolution [20], declining extinction rates [9], [21], the ‘Tangled Nature’ model of macroevolution [4], [10], fluctuating commodity prices [22], type-II superconductors [11], colloidal gels [14], and spin glasses [8]. To our knowledge this is the first experimental evidence for record dynamics in living systems, that is, those that have been shaped by Darwinian evolution.

The concept of complex systems was developed in the physical sciences to explain the emergence of macroscopic phenomena from the interactions of large numbers of microscopic components. In biology this approach has greatly aided our understanding of collective phenomena such as decentralised control and pattern formation [23]. The theory of self-organisation was originally developed to explain pattern formation in stationary physical systems - those at a statistical steady state. However the physical systems that decelerate according to record dynamics are manifestly non-stationary. The identification of the record signal even in purely physical systems is a hard problem, indeed the only case in which the record signal has been unequivocally identified (i.e. thermal energy) is the Edwards-Anderson spin-glass [4].

We have described a biological social system that displays the same statistics as found in many non-stationary physical systems governed by decelerating record dynamics, and hence infer that a similar statistical mechanism is in operation in the ants. What could be the individual mechanism underlying the collective record dynamics? One plausible scenario is that individual variation in the perception of the record signal can lead to variation in the memory of the standing record amongst individuals. We have demonstrated that decision-making in ant societies may originate from the combination of fluctuating record signals and ant-ant interactions. This suggests that further understanding of signals and interactions between individuals within the colony could elucidate not only the organisation of insect societies but also facilitate the understanding of general principles of system organisation. The challenge for biologists is to identify these signals and interactions by quantifying the behaviour of the individual colony members over time. This opens a future avenue for new manipulative experimentation and theory.

## Supporting Information

Text S1 Electronic supplementary material. Refers to Table S1, and Figures S1, S2 and S3.
(0.32 MB PDF)

Table S1 Regression statistics for the individual colonies. Least squares linear regressions were performed on the survivorship plots, P(*τ*>x), for *τ* = Ln(T_k_)-Ln(T_k-1_) (Fig. 3 and Fig. S1 a–f).
(0.05 MB DOC)

Figure S1 Logarithmic waiting times are exponentially distributed for two-hour removals. Survivorship of the logarithmic waiting times, τ, that is P(*τ*>x). a–f) The six colonies that underwent a two-hour removal. g) Cumulative distribution for the scaled waiting times for the same data. Solid line- empirical data. Circles- Fit provided by the scaled waiting time distribution expected from a Log-Poisson process; P (( T_k_ - T_k-1_)/T_k-1_ <x ) = 1-(x+1)^−*α*^. Here the best fit was provided by *α* = 13.
(0.11 MB TIF)

Figure S2 The time-series of logarithmic waiting times is ‘memoryless’. Mean autocorrelation of the logged ratio between successive exit times, τ, and the lagged values.
(0.04 MB PDF)
