# Supplementary material for: Record Dynamics in Ants
Source: PLoS One. 2010 Mar 11;5(3):e9621. doi: 10.1371/journal.pone.0009621 (PMC2836372; doi:10.1371/journal.pone.0009621)
Supplement: Text S1 — Electronic supplementary material. Refers to Table S1, and Figures S1, S2 and S3. (0.32 MB PDF) [file pone.0009621.s001.pdf]

Electronic Supplementary Material

Poisson statistics have many well-studied properties. Here we present extended analyses of the distribution of logarithmic waiting times for six colonies undergoing a short two hour removal (Fig. S1). We then show an additional demonstration of the absence of temporal correlations in the time-series of logarithmic waiting times, expected for log-Poisson record dynamics (Fig. S2). The details of the least-squares linear regressions used to parameterise the logarithmic waiting time distributions are also included (Table S1). Finally, we present the empirical distribution of gaster weights (Fig. S3) used to parameterise the null model.

The ratio between the logarithms of sequential exit times is exponentially distributed.
The difference between the logarithms of successive exit times, τ, can be used to recognise log-Poisson processes [1]. In figure. S1a-g we show the τ distributions for the six colonies (i-iv) that underwent a shorter two hour removal. These are the data that were pooled to produce Fig. 3 (column 1, row 2) in the main paper. It can be seen that when plotted separately the τ distributions are still exponential. In a log-Poisson process, the probability that an event occurs *within* a time x, is [1,2]:

$$P\left(\left(T_k - T_{k-1}\right) / T_{k-1} < x\right) = 1 - (x+1)^{-\alpha}$$

The left hand-side of the above equation is the observed cumulative distribution of scaled waiting times. The right hand-side is the prediction from relaxation in condensed-matter systems, and here α is expected to be >1 [1,2]. If the agreement between the right and left hand-sides of the above equation is reasonable, a log-Poisson process may be inferred. We found that the cumulative distribution of scaled waiting times for the short removal period is in good agreement with this prediction (Fig. S1g.)

Successive events occur independently.
There is little correlation between in the time-series of successive logarithmic waiting times, τ (Fig S2). Indeed the longest observation periods, for both removal and non-removal conditions showed the least correlation, so the exit statistics can be said to be 'memoryless'.

References

1. Sibani P (2005) Extremal noise events, intermittency and Log-Poisson statistics in non-equilibrium aging of complex systems. Proc SPIE 5845 271: 271-282.
2. Sibani P, Dall J (2003) Log-Poisson statistics and full aging in glassy systems. Europhys Lett 64: 8-14.
