# Supplementary material for: Record Dynamics in Ants
Source: PLoS One. 2010 Mar 11;5(3):e9621. doi: 10.1371/journal.pone.0009621 (PMC2836372; doi:10.1371/journal.pone.0009621)
Supplement: Table S1 — Regression statistics for the individual colonies. Least squares linear regressions were performed on the survivorship plots, P(τ>x), for τ = Ln(Tk)-Ln(Tk-1) (Fig. 3 and Fig. S1 a–f). (0.05 MB DOC) [file pone.0009621.s002.doc]

|  |  |  | *Slope* | | | *Residuals* | |
| --- | --- | --- | --- | --- | --- | --- | --- |
| Removal/Non-Removal | Colony | N exits | R^2^ (adj) | p | λ | Anderson-Darling | p |
| R | i | 39 | 96.40% | <0.0001 | 4.54 | 0.251 | 0.714 |
| R | ii | 44 | 94.80% | <0.0001 | 6.41 | 0.251 | 0.079 |
| R | iii | 30 | 96.40% | <0.0001 | 4.77 | 0.729 | 0.049 |
| R | iv | 63 | 95.90% | <0.0001 | 5.8 | 2.717 | <0.005 |
| R | v | 20 | 97.50% | <0.0001 | 3.48 | 0.372 | 0.38 |
| R | vi | 39 | 99.30% | <0.0001 | 8.25 | 1.104 | 0.006 |
| R | 1 | 47 | 94.80% | <0.0001 | 2.49 | 0.59 | 0.11 |
| R | 2 | 40 | 93.80% | <0.0001 | 1.59 | 0.19 | 0.89 |
| R | 5 | 18 | 94.60% | <0.0001 | 0.59 | 0.27 | 0.55 |
| R | 6 | 40 | 92.90% | <0.0001 | 2.22 | 1.06 | 0.007 |
| R | 7 | 14 | 94.80% | 0.003 | 0.66 | 0.52 | 0.09 |
| R | 8 | 96 | 97.40% | <0.0001 | 3.35 | 0.99 | 0.012 |
| R | 9 | 77 | 98.10% | <0.0001 | 3.59 | 0.466 | 0.247 |
| NR | 1 | 40 | 90.60% | <0.0001 | 3.03 | 0.792 | 0.038 |
| NR | 2 | 21 | 96.00% | <0.0001 | 3.33 | 0.736 | 0.051 |
| NR | 3 | 26 | 96.00% | <0.0001 | 1.17 | 0.525 | 0.156 |
| NR | 4 | 45 | 97.80% | <0.0001 | 2.23 | 0.249 | 0.729 |
| NR | 5 | 43 | 92.60% | <0.0001 | 2.52 | 0.617 | 0.101 |
| NR | 6 | 46 | 89.60% | <0.0001 | 1.98 | 1.14 | <0.005 |
| NR | 7 | 82 | 97.50% | <0.0001 | 2.44 | 0.872 | 0.021 |
