# Supplementary figures and images for: Record Dynamics in Ants
Source: PLoS One. 2010 Mar 11;5(3):e9621. doi: 10.1371/journal.pone.0009621 (PMC2836372; doi:10.1371/journal.pone.0009621)

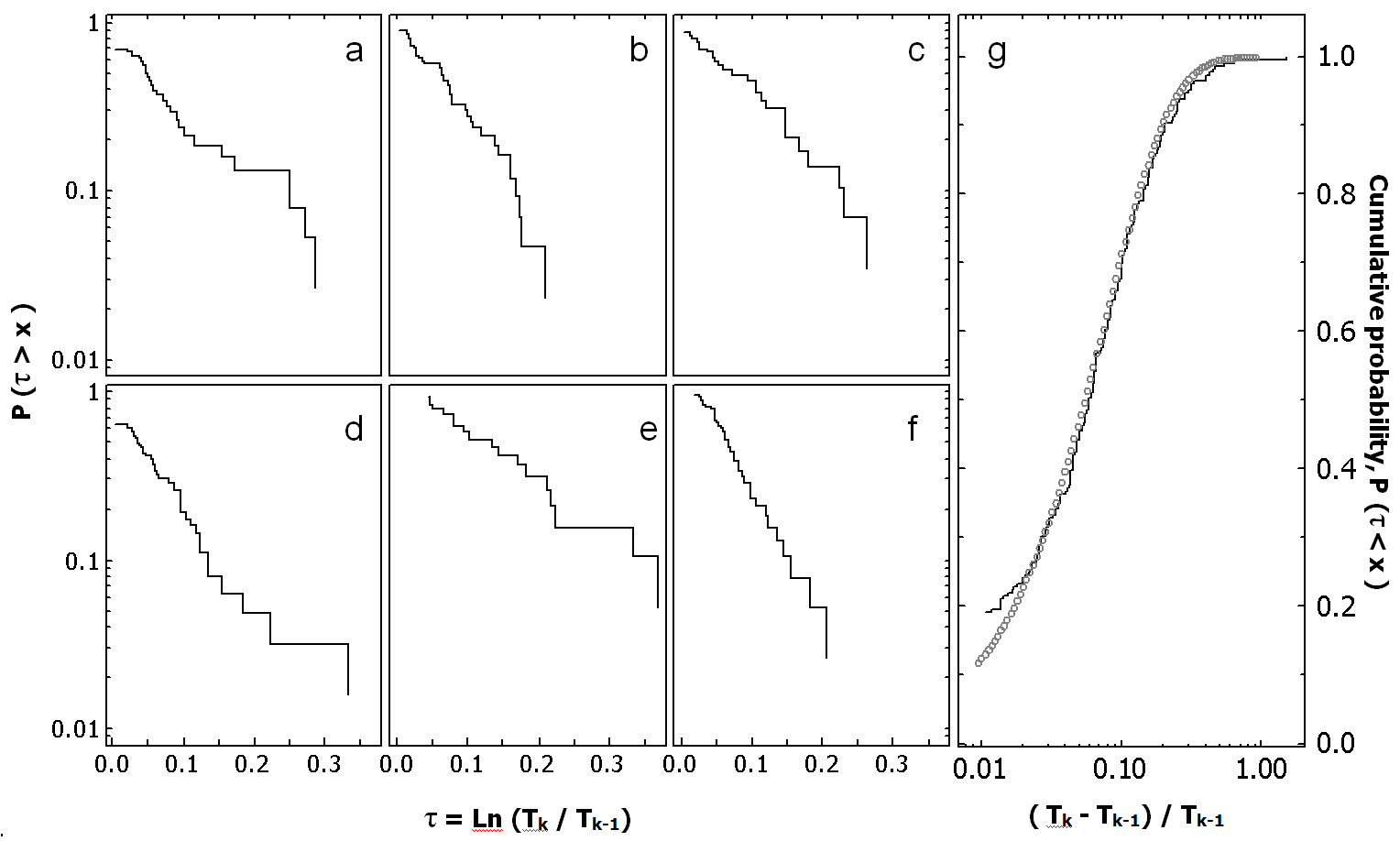

Supplement: Figure S1 — Logarithmic waiting times are exponentially distributed for two-hour removals. Survivorship of the logarithmic waiting times, τ, that is P(τ>x). a–f) The six colonies that underwent a two-hour removal. g) Cumulative distribution for the scaled waiting times for the same data. Solid line- empirical data. Circles- Fit provided by the scaled waiting time distribution expected from a Log-Poisson process; P (( Tk - Tk-1)/Tk-1 <x ) = 1-(x+1)−α. Here the best fit was provided by α = 13. (0.11 MB TIF) [file pone.0009621.s003.tif]

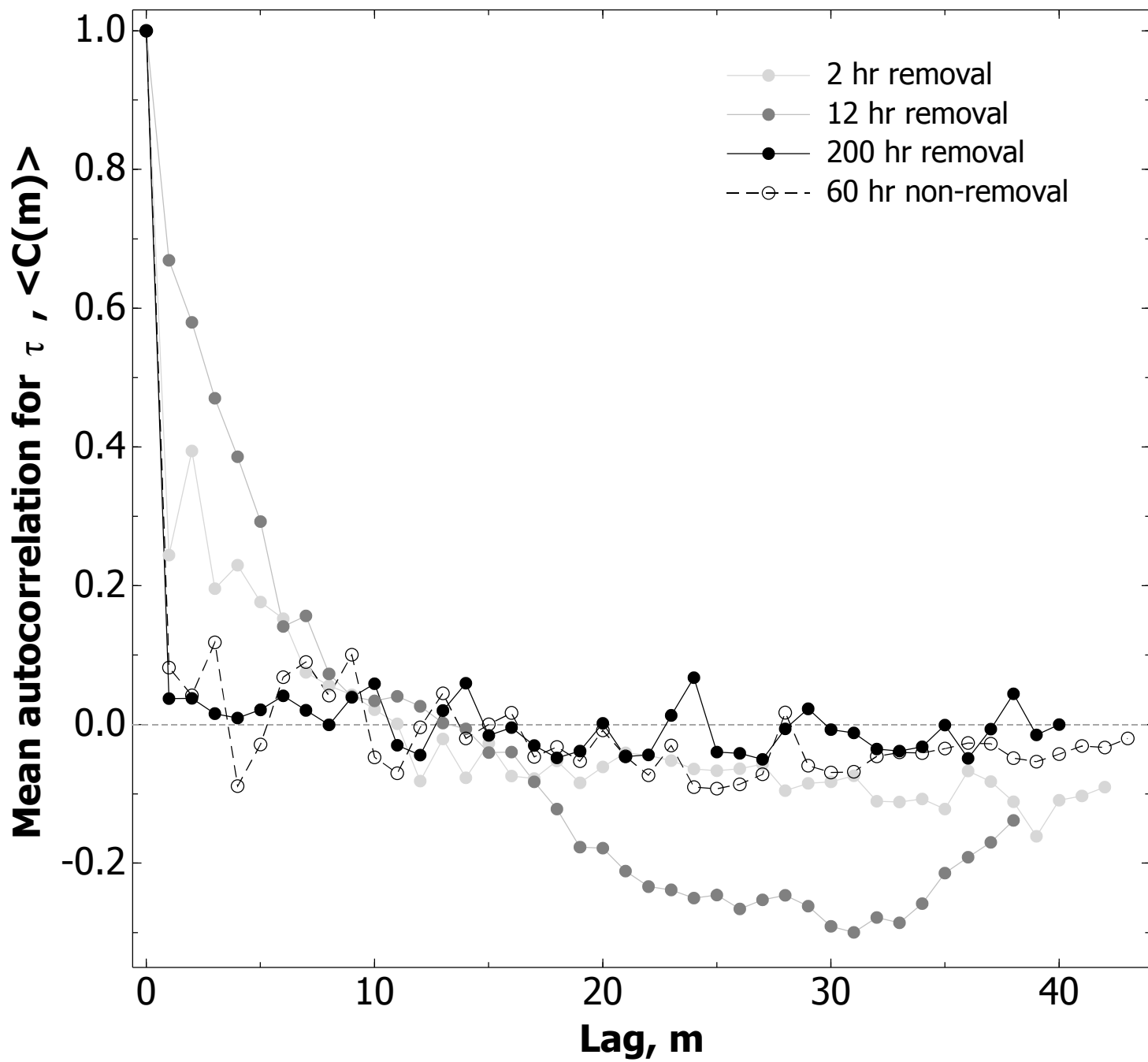

Supplement: Figure S2 — The time-series of logarithmic waiting times is ‘memoryless’. Mean autocorrelation of the logged ratio between successive exit times, τ, and the lagged values. (0.04 MB PDF) [file pone.0009621.s004.pdf]
